# Time trends in the mortality of testicular cancer across the BRICS: an age-period-cohort analysis for the GBD 2019

**DOI:** 10.1038/s41598-024-63191-9

**Published:** 2024-06-03

**Authors:** Yuting Xu, Shudong Xie, Chengyu Zhou, Liping Zhu, Yao Tong, Alvaro Munoz, Yuhang Wu, Xuhong Li

**Affiliations:** 1 Department of Rehabilitation Medicine, The Third Xiangya Hospital, Central South University, Changsha, Hunan China; 2 Transplantation Center, The Third Xiangya Hospital, Central South University, Changsha, Hunan China; 3 Centro Universitario del Norte, Universidad de Guadalajara, Colotlán, Jalisco Mexico; 4 Department of Epidemiology and Health Statistics, Xiangya School of Public Health, Central South University, Changsha, Hunan China

**Keywords:** Testicular cancer, Mortality, Age-period-cohort, Trend, Cancer, Environmental social sciences, Diseases, Health care, Medical research

## Abstract

Testicular cancer (TCa) is a rare but impactful malignancy that primarily affects young men. Understanding the mortality rate of TCa is crucial for improving prevention and treatment strategies to reduce the risk of death among patients. We obtained TCa mortality data by place (5 countries), age (20–79 years), and year (1990–2019) from the Global Burden of Disease Study 2019. Age-period-cohort model was used to estimate the net drift, local drift, age effects, period and cohort effects. In 2019, the global mortality of TCa increased to 10842 (95% UI 9961, 11902), with an increase of 50.08% compared to 1990.The all-age mortality rate for TCa in 2019 increased from 0.17/100,000 (95% UI 0.13, 0.20) in China to 0.48/100,000 (95% UI 0.38, 0.59) in Russian Federation, whereas the age-standardized mortality rate in 2019 was highest in the South Africa 0.47/100,000 (95% UI 0.42, 0.53) and lowest in the China 0.16/100,000 (95% UI 0.13, 0.19). China's aging population shifts mortality patterns towards the elderly, while in Russian Federation, young individuals are primarily affected by the distribution of deaths. To address divergent TCa mortality advancements in BRICS countries, we propose a contextually adaptive and resource-conscious approach to prioritize TCa prevention. Tailoring strategies to contextual diversity, including policy frameworks, human resources, and financial capacities, will enhance targeted interventions and effectiveness in reducing TCa mortality.

## Introduction

Testicular cancer (TCa) is the third leading cause of cancer deaths and the predominant malignancy in young men aged 15–45, constituting 1% of male cancers and 5% of urologic malignancies^1,2^. Despite a global increase in incidence, the mortality rate has been declining, primarily due to the introduction of platinum-based chemotherapy in the 1970s^3–7^. However, Platinum agents cause DNA damage, resulting in the production of reactive platinum in the bloodstream and the formation of platinum-DNA adducts in organs, which can be detected even after 20 years following chemotherapy^8^. This further leads to complications associated with platinum-based drugs, including secondary malignant neoplasms (SMN), cardiovascular disease (CVD), and neurotoxicity^9^. To better understand the prognostic and survival outcomes associated with TCa, examining the ongoing burden of TCa-associated mortality is critical to public health policy development.

TCa has a high mortality rate in developing countries, and its study can serve as a tool to support the planning of public health policies for the most vulnerable groups^10^. The BRICS countries, comprising Brazil, the Russian Federation, India, China, and South Africa, belong to the developing world and are marked by their rapid economic development, forming a strong political and economic alliance that represents nearly half of the world's population^11^. These nations are increasingly allocating a significant portion of their economic resources to healthcare expenditures. However, they still face significant challenges in balancing the diverse needs of promoting public health, controlling non-communicable diseases, and improving population health^12^. Therefore, studying the BRICS countries is vital to comprehend the disease's impact on a large global population and develop targeted strategies for promoting public health and controlling diseases in these nations.

Previous studies have examined global trends in TCa incidence, mortality, and disability-adjusted life-years (DALYs) using data from the 2016 Global Burden of Disease study (GBD), primarily focusing on age-standardized rates. However, these overall rates may mask specific factors that contribute significantly to the disease burden, posing challenges in comprehensively analyzing the temporal trends of TCa^13^. This study visualized global and BRICS TCa mortality data by age, year, and region for the period 1990–2019 to characterize the most up-to-date global epidemiology of TCa, updating the results of previous studies on the burden of TCa mortality. Age-period-cohort (APC) model was then used to further examine the effects of age, period, and birth cohort effects on changes in TCa mortality over the previous three decades. Changes in disease patterns can be illustrated by the findings from this study, shedding light on how resources should be allocated and providing guidance for the development of prevention and control strategies to further lessen the burden of TCa.

## Materials and methods

### Data sources

The data utilized in this study were acquired from the Global Health Data Exchange GBD Results Tool (https://vizhub.healthdata.org/gbd-results/). The GBD 2019 study offers the latest and comprehensive descriptive epidemiological data for 369 diseases and injuries across 204 countries and territories from 1990 to 2019^14,15^. We obtained the mortality numbers, all-age mortality rates, and age-standardized mortality rates of TCa for males globally and in BRICS countries, across age groups from 20 to 79 years old, spanning the years 1990 to 2019. All estimates were reported in 95% uncertainty intervals (UIs), which were sampled repeatedly 1000 times, with upper and lower bounds based on the 2.5th and 97.5th percentiles of the uncertainty distribution^14^. The available data used in this study were anonymous and publicly accessible, and the University of Washington Institutional Review Board granted approval for a waiver of informed consent. TCa diagnoses and classifications in this study were based on the clinical criteria outlined by the World Health Organization (WHO), as well as the International Statistical Classification of Diseases (ISCD) and the International Classification of Diseases and Injuries (ICD-10). The specific ICD-10 codes used for TCa were C62.0, C62.1, and C62.9 available from https://icd.who.int/browse10/2019/en.

### Statistical analysis

#### Age–period–cohort analysis

The APC model was employed in this study to analyze the data, incorporating age, period, and cohort as independent variables. The model treated the incidence of the observed event or phenomenon in the population as the dependent variable, assuming a probability distribution. By utilizing APC models, this study goes beyond traditional epidemiological analyses to uncover the influence of various factors on disease trends^15^. In the APC model, the age effect represents the risk of different outcomes in different age groups, the period effect reflects the change in outcomes over time, affecting all age groups at the same time, and the cohort effect reflects the change in outcomes among people with the same year of birth^16^.

#### Data arrangement

In order to control the number of parameters in the APC model while obtaining a smooth time-effects curve, age-specific mortality rates in this study were divided into 5-year groups at equal intervals (20–24, 25–29, ..., 75–79 years). The age groups below 20 years old and above 80 years old were not included in this study due to the absence or rarity of TCa events or TCa-related deaths. In a standard APC model, it is customary for the age and period intervals to be of equal length. For instance, five-year age groups are typically used alongside five-year calendar periods. However, in this study, the GBD data was integrated into a unified framework by extracting the mortality and population counts from the mid-year of six specific time points (1992, 1997, ..., 2017) instead of using the five-year averages to represent the corresponding periods. As birth cohorts are defined by the age of the subjects and the dates of event occurrence, that is, cohort = period - age^17^, the corresponding birth cohorts ranged from 1911–1919 (median year 1915) to 1991–1999 (median year 1995). The lexis diagram of GBD data for the APC model was shown in Additional Table 1.

**Table 1:** Trends in the mortality of TCa among male globally and in the BRICS countries, 1990–2019.

	Global		Brazil		China		India		Russian Federation		South Africa	
	1990	2019	1990	2019	1990	2019	1990	2019	1990	2019	1990	2019
Population												
Number, n × 1,000,000	5350 (5329, 5460)	7737 (7483, 7993)	149 (138, 159)	217 (190, 243)	1184 (1103, 1272)	1422 (1239, 1597)	856 (792, 919)	1391 (1238, 1559)	151 (139, 163)	147 (129, 165)	37 (33, 41)	56 (49, 63)
Percentage of global, %	100.00	100.00	2.79	2.80	22.13	18.38	16.00	17.98	2.82	1.90	0.69	0.72
Deaths												
Number	7224 (6792, 7906)	10842 (9961, 11902)	233 (215, 304)	449 (418, 488)	782 (650, 908)	1205 (964, 1465)	1020 (884, 1170)	1704 (1398, 2036)	424 (387, 448)	327 (262, 401)	56 (45, 66)	92 (81, 104)
Percentage of global, %	100.00	100.00	3.23	4.14	10.83	11.11	14.12	15.72	5.87	3.02	0.78	0.85
Percent change of deaths 1990–2019, %	50.08 (36.78, 64.77)		92.20 (49.10, 114.14)		54.09 (15.17, 101.54)		67.08 (29.11, 112.36)		− 22.76 (− 35.97, − 3.61)		64.41 (40.32, 95.33)	
All-age mortality rate												
Rate per 100,000	0.27 (0.25, 0.29)	0.28 (0.26, 0.31)	0.32 (0.29, 0.41)	0.42 (0.40, 0.46)	0.13 (0.11, 0.15)	0.17 (0.13, 0.20)	0.23 (0.20, 0.26)	0.24 (0.20, 0.29)	0.60 (0.55, 0.63)	0.48 (0.38, 0.59)	0.32 (0.26, 0.37)	0.34 (0.30, 0.38)
Percent change of rate 1990−2019, %	4.17 (− 5.06, 14.36)		33.65 (3.68, 48.90)		29.72 (− 3.05, 69.67)		4.33 (− 19.38, 32.61)		− 20.04 (− 33.72, − 0.22)		7.37 (− 8.36, 27.56)	
Age-standardized mortality rate												
Rate per 100,000	0.31 (0.29, 0.33)	0.28 (0.26, 0.31)	0.37 (0.35, 0.46)	0.41 (0.38, 0.45)	0.16 (0.13, 0.18)	0.16 (0.13, 0.19)	0.27 (0.23, 0.31)	0.24 (0.20, 0.29)	0.63 (0.58, 0.66)	0.42 (0.34, 0.51)	0.52 (0.39, 0.61)	0.47 (0.42, 0.53)
Percent change of rate 1990−2019, %	− 8.81 (− 16.96, − 0.24)		9.12 (− 8.55, 19.72)		− 1.62 (− 24.16, 26.91)		− 10.10 (− 30.66, 15.27)		− 32.98 (− 44.39, − 16.31)		− 8.02 (− 21.23, 15.57)	
APC model estimates												
Net drift of mortality rate, % per year	− 0.49 (− 0.62, − 0.36)		0.20 (− 0.53, 0.93)		− 0.69 (− 1.06, − 0.32)		− 0.56 (− 0.96, − 0.15)		− 1.90 (− 2.45, − 1.35)		− 0.63 (− 1.95, 0.71)	

All-age mortality rate = crude mortality rate. Age-standardized mortality rate is computed by direct standardization with global standard population in GBD 2019. Net drifts are estimates derived from the age-period-cohort model and denotes overall annual percentage change in mortality, which captures the contribution of the effects from calendar time and successive birth cohorts. Parentheses for all GBD health estimate indicate 95% uncertainty intervals; parentheses for net drift indicate 95% confidence intervals.

*APC* age-period-cohort.

In this study, our primary focus lies on the following estimable functions. The net drift captures the overall annual percentage change in mortality rates over time. Local drifts elucidate the annual percentage changes by period and cohort for each age group. The longitudinal age curve illustrates the fitted age-specific rates over time in the reference cohort, accounting for period deviations. The period (or cohort) rate ratio (RR) denotes the ratio of age-specific rates in each period (or cohort) compared to the reference period (or cohort). A RR value > 1 implies increased risk of TCa mortality, while a RR value < 1 suggests decreased risk. In our study, we used the intrinsic estimator (IE) method associated with the APC model to address the identification problem caused by linear relationships between age, period, and cohort, thereby overcoming the unpredictability of model parameters. Further details regarding the methodology can be found in previous literature^18^.The significance of the estimated parameters and functions was assessed using the Wald chi-squared test, with all statistical tests conducted as two-tailed. The Age Period Cohort (APC) analysis in this study was performed utilizing the APC Web Tool provided by the National Cancer Institute (http://analysistools.nci.nih.gov/apc/). All graphical visualizations were created using the R statistical program (version 4.0.3).

### Ethical information

Data were all analyzed anonymously, so ethical approval was not needed. All methods in this paper were performed following the relevant guidelines and regulations.

## Results

### Globally and in BRICS countries trends in TCa mortality, 1990-2019

Table 1 shows the population, total number of deaths, all age mortality rate, age-standardized mortality rate, and net drift of mortality. Over the past three decades, the number of TCa deaths increased from 7224 (95% UI 6792, 7906) in 1990 to 10,842 (95% UI 9961, 11,902) in 2019, representing a 50.08% increase. The age-standardized mortality rate decreased from 0.31 (95% UI 0.29, 0.33) per 100,000 population in 1990 to 0.28 (95% UI 0.26, 0.31) per 100,000 population in 2019, indicating an 8.81% decrease. The APC model estimated a net drift of − 0.49 (95% CI − 0.62, − 0.36) in TCa mortality from 1990 to 2019 globally (Table 1).

In the BRICS countries, the Russian Federation witnessed a declining proportion of global TCa mortality from 1990 to 2019, while Brazil, China, India, and South Africa observed an increasing proportion. In 2019, the all-age mortality rate for TCa ranged from 0.17 (95% UI 0.13, 0.20) per 100,000 population in China to 0.48 (95% UI 0.38, 0.59) per 100,000 population in the Russian Federation. The age-standardized mortality rate in 2019 was highest in South Africa at 0.47 (95% UI 0.42, 0.53) per 100,000 population and lowest in China at 0.16 (95% UI 0.13, 0.19) per 100,000 population. In Brazil, there has been an upward trend in the age-standardized mortality rate of TCa from 1990 to 2019, reaching 9.12 (95% UI − 8.55, 19.72). Conversely, the other BRICS countries have shown a declining trend. Among these nations, the Russian Federation exhibited the most significant decrease, with a rate of − 32.98 (95% UI − 44.39, − 16.31), while China had the least significant decrease, with a rate of − 1.62 (95% UI − 24.16, 26.91). According to the APC model estimates, the annual net drift in TCa mortality ranged from − 1.90 (95% UI − 2.45, − 1.35) in the Russian Federation to 0.20 (95% UI − 0.53, 0.93) in Brazil within the BRICS countries (Table 1).

### Time trends in TCa mortality across different age groups

**Figure 1:**
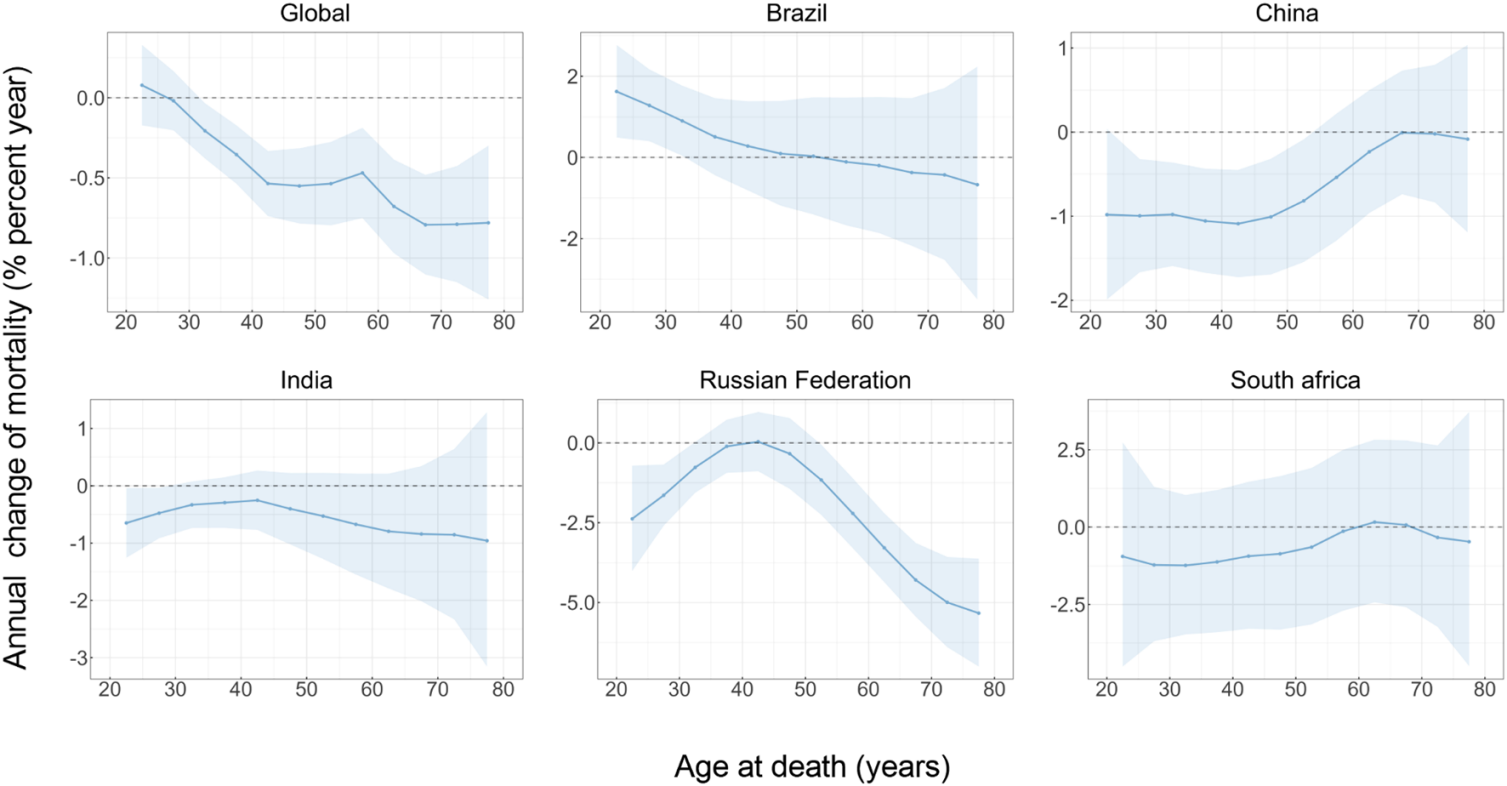
The local drifts of TCa mortality rate in global and BRICS, 1990–2019. Local drifts of TCa mortality rate (estimates from age-period-cohort models) for 12 age groups (20–24, 25–29, …, 75–79 years), 1990–2019. The dots and shaded areas indicate the annual percentage change of incidence rate (% per year) and the corresponding 95% CIs.

Figure 1 shows the annual percentage change of mortality rate of TCa in different age groups. Globally, the majority of age groups exhibited values of local drift that were predominantly below 0, suggesting a decline in the mortality rate of TCa for most age groups, except for the younger age group (20–24 years). A similar trend was observed in Brazil, but with a broader range of age groups experiencing an increase in mortality, particularly among the younger age group (20–54 years). It is noteworthy that a decline in TCa mortality rates has been observed across almost all age groups in China, India, Russian Federation, and South Africa.

**Figure 2:**
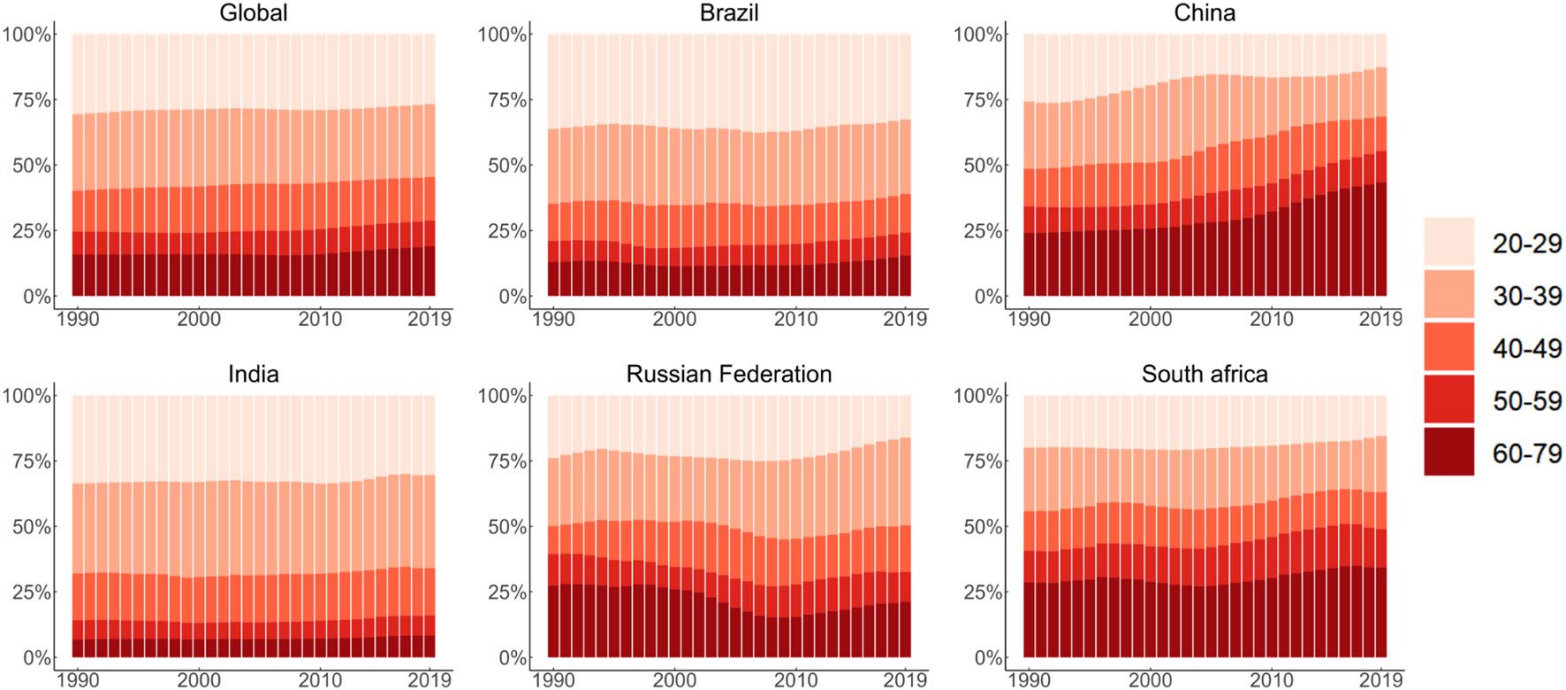
Age distribution of deaths from TCa in global and BRICS, 1990-2019. Age distribution of deaths is represented as temporal change in the relative proportion deaths across age groups (20–24, 25–29, …, 75–79 years) during 1990–2019.

Figure 2 presents temporal changes in the age distribution of deaths. From 1990 to 2019, the global proportion of deaths due to TCa remained stable across various age groups, and similar patterns were observed in Brazil, India, and South Africa. However, in China, the distribution of deaths from TCa showed a pattern of transition from the lower age group (20–39 years) to the higher age group (≥ 60–79 years). In Russian Federation, the age distribution of deaths primarily impacts young individuals within specific age ranges (20–24, 25–29, 30–34, 35–39 years).

### Age, period, and cohort effects on TCa mortality

**Figure 3:**
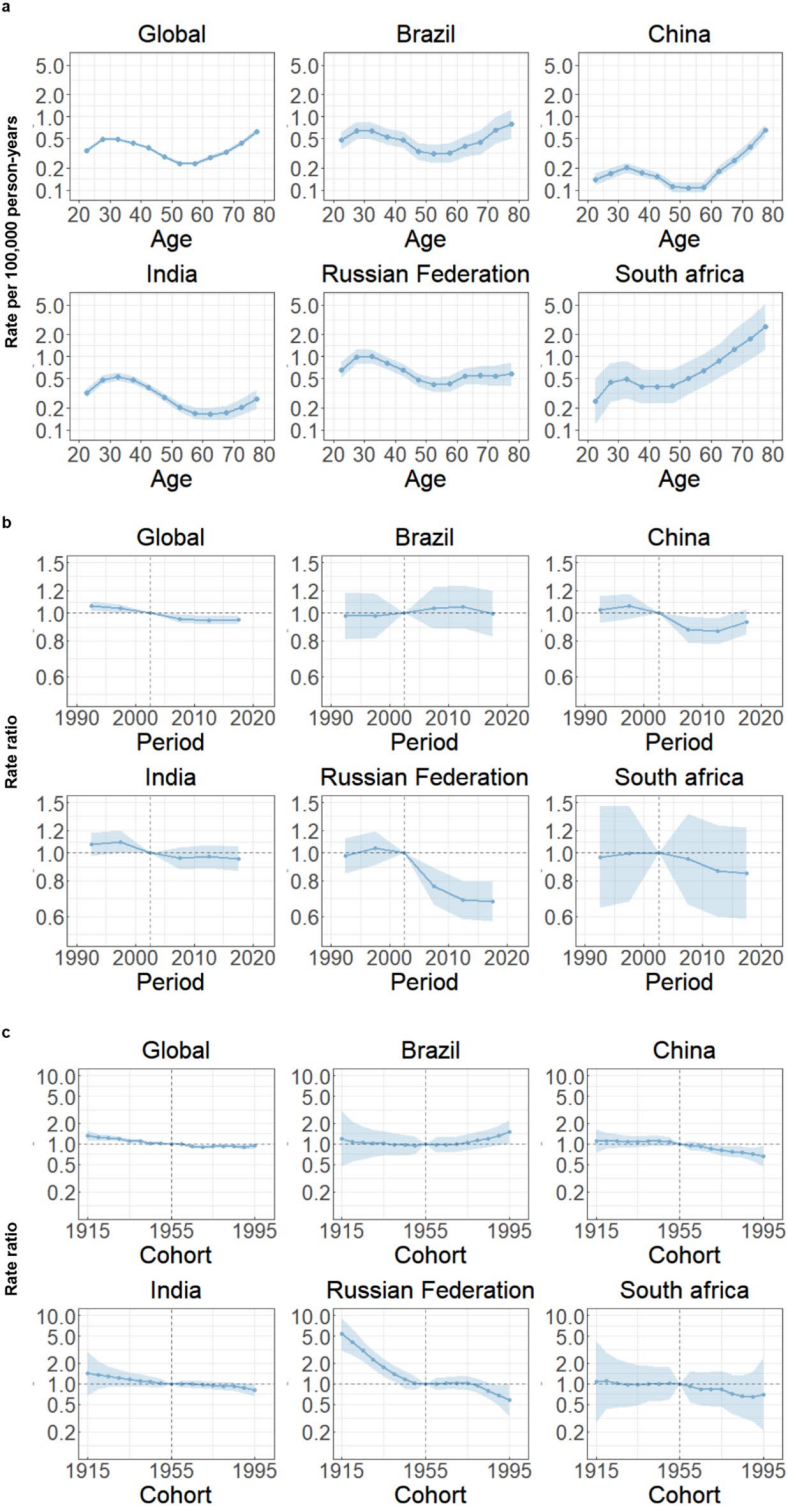
Age, period and cohort effects on TCa mortality in global and BRICS. (**a**) Age effects are shown by the fitted longitudinal age curves of mortality rate (per 100,000 person-years) adjusted for period deviations. (**b**) Period effects are shown by the relative risk of mortality rate (mortality rate ratio) and computed as the ratio of age-specific rates from 1990–1994 to 2015–2019, with the referent cohort set at 2000–2004. (**c**) Cohort effects are shown by the relative risk of mortality rate and computed as the ratio of age-specific rates from the 1915 cohort to the 1995 cohort, with the referent cohort set at 1955. The dots and shaded areas denote mortality rates or rate ratios and their corresponding 95% CIs.

Figure 3a shows the Age-period-cohort effects estimates derived from the APC model by global and BRICS countries. Globally, within the 25–34 age group, the highest risk was observed among younger individuals, followed by a gradual decline in risk with increasing age. The lowest risk was found in the 50–59 age group, followed by a continuous increase in risk. This suggests that young individuals are more susceptible to the life-threatening effects of TCa, and with the influence of aging on the body, the mortality risks accumulated during youth may resurface in later years. Similar patterns were observed in Brazil. Contrary to global patterns, Russian Federation and India demonstrated a slow increase in mortality risk among the older age group (60 years and above), while the highest mortality risk was observed in the younger age group (25–34 years). Notably, China and South Africa showed relatively stable mortality rates within the age group of 20–59 years, with an increase in mortality risk observed in the age group above 60 years (Fig. 3a)

Globally, the period effects remained relatively consistent over the past three decades, indicating limited improvement in mortality risk. Similar patterns were observed in Brazil. However, there have been varying degrees of decline in mortality risk in China, India, Russian Federation, and South Africa compared to the reference period (Fig. 3b).

Globally, cohort effects showed a slight decrease in the successive birth cohort. Similar patterns were observed in China, South Africa. The risk of cohort effects increases with birth cohort in Brazil, especially after 1970, this risk increases with birth cohort, while in India and the Russian Federation it remained stable. (Fig. 3c).

## Discussion

To our knowledge, this is the first study to analyze TCa mortality using the APC model, enabling comparisons between global and BRICS countries. Our study’s main contribution, compared to previous GBD 2016 publications^13^, lies in providing a more comprehensive understanding of disease trends and utilizing age, period, and cohort effects to distinguish between different sources of mortality risk on a global scale as well as within the BRICS countries. Another significant advancement is the measurement of localized drift in the global and BRICS age distributions of mortality and age at onset from 1990 to 2019. This approach allows us to capture temporal trends in incidence for each age group and account for period effects. Our study, incorporating the APC model, offers valuable insights for policymakers and healthcare providers by providing a nuanced analysis of TCa mortality and assessing the effectiveness of related healthcare services.

Between 1990 and 2019, the total number of TCa deaths worldwide increased by 50.08% (95% UI 36.78, 64.77), likely driven by population growth. Among BRICS, Brazil, China, India, and South Africa experienced varying degrees of increase, while Russian Federation saw a decrease. In particular, some of the information changed differently after eliminating inconsistencies in age composition, with global age-standardized TCa mortality declining over this 30-year period. This is largely attributed to the introduction of cisplatin-based chemotherapy in the 1970s^7^. However, the use of cisplatin-based chemotherapy is associated with significant side effects, including potentially fatal outcomes^8,9^. Therefore, addressing the burden of this disease requires further attention and consideration. The distribution of TCa deaths, which remains relatively stable in the majority of age groups across the global and BRICS countries (including Brazil, India, and South Africa), which can be attributed to increased awareness of TCa, wider use of ultrasound at the grass-roots level, as well as centralization of treatment and development of guidelines^19,20^. Population aging in China has resulted in a transition of mortality characteristics from younger individuals to the middle-aged and elderly population^21^, whereas in Russian Federation, the affected group primarily consists of young people influenced by the distribution of deaths^22,23^.

In China and South Africa, they exhibit significant similarities in terms of age, period, and cohort. There has been an improvement in the mortality rate among consecutive cohorts of younger birth. The decline in mortality in China and South Africa was an important contributor to the slight reduction in mortality risk over the global period and cohort. These advances likely stemmed from the establishment of the first cancer service in China in 1959, dedicated to cancer prevention and control^24^. This has increased the rate of early detection of TCa, thereby helping to reduce the mortality rate from TCa. China's "12th Five-year Health Plan" (2011–2015) and the "Healthy China 2030" blueprint (2016) aimed to expand healthcare coverage, increase government investment, and reduce the financial burden of medical expenses^25,26^. These initiatives have improved access to medical services for TCa patients. Similarly, the collection and analysis of cancer statistics by the South African National Cancer Registry (SANCR) contribute to the reduction of mortality risk in South Africa^27^. Despite these advancements, both China and South Africa still have room for improvement as they face high mortality risks among individuals aged over 60. China and South Africa were undergoing demographic transitions characterized by population aging and an increase in average life expectancy^28,29^. This transition has led to an elevated mortality of malignant tumors among the elderly population.

Russian Federation and India also share similarities in terms of age, period and cohort effects. The highest risk of TCa mortality is observed among males aged 25-34. The popularity of overweight and obesity, which tends to be younger in India and Russian Federation, is considered to be a public health problem that seriously affects the health of the population^30–32^. It is encouraging to observe a significant decline in period effects in both India and Russian Federation. In India, this can be attributed to the implementation of the National Cancer Control Programme launched in 1975^33^. The program focused on primary prevention through health education, secondary prevention through early detection and diagnosis, support for existing cancer treatment facilities, and improvements in end-of-life care provision^34^. In Soviet era, mandatory annual clinical examinations were an integral part of the lives of Soviet citizens^35^. Since 2013, mandatory health insurance in contemporary Russian Federation has ensured the continuity of clinical examinations, which includes cancer-related screening as an important component^35^, potentially contributing to the decline in cancer mortality in the country. The confluence of detrimental factors associated with obesity and favorable factors, such as diverse cancer screening policies, may have contributed to the stabilization of cohort dynamics observed over the past three decades in India and Russian Federation.

In Brazil, individuals aged 25–34 and the elderly have the higher mortality risk. The environmental changes and exposure to environmental endocrine disruptors (EEDs) endocrine-disrupting chemicals (EEDs) associated with industrialization and urbanization may be closely related to the development of TCa^36^. Although the specific causal relationship requires further research, the process of industrialization plausibly help explain the higher mortality risk among young people. And a significant proportion of older persons report difficulties in accessing health-care services when needed in Brazil^37^, which could potentially be relevant to the increased risk of death from TCa in the older population. The cohort-based mortality risk of TCa in Brazil has significantly increased in recent decades, potentially due to the impact of pesticide use. As one of the largest consumers of pesticides globally, Brazil plays a major role in the international pesticide market^38^. The usage of pesticides in Brazil has been linked to an elevated risk of mortality for testicular cancer^39^.

Admittedly, several limitations should be acknowledged: (I) This study lacks a more granular analysis to capture subnational differences, as there are variations in health issues and access to healthcare providers and services at the subnational level; (II) Despite significant efforts in data standardization, the uncertainty related to data quality control, including data collection procedures, handling, and coding, remains unavoidable. Variations in data collection methods across different regions or healthcare systems may introduce bias or measurement error, potentially impacting the accuracy and comparability of the reported trends. Thus, the findings should be interpreted with caution, considering the potential influence of data quality variation on the observed associations or trends; (III) The utilization of five-year age intervals, which is the most commonly used data format in the APC model, may lead to the attenuation of minor variations in age, period, and cohort effects; (IV) In this analysis, period and cohort effects were examined based on estimated cross-sectional data from the Global Burden of Disease study spanning from 1990 to 2019. However, future cohort studies in different countries are needed to assess location- and time-specific relative risks and evaluate diverse risks among vulnerable populations.

## Conclusions

The BRICS countries have made varying levels of progress in reducing TCa mortality. We acknowledge the diverse environmental contexts within the BRICS countries and recommend a gradual advancement of TCa prevention efforts based on specific circumstances, utilizing available policy-driven, human, and financial resources. To improve the prevention and treatment of TCa, healthcare services should be expanded to encompass males of all age groups, with particular attention given to vulnerable populations.

### Supplementary Information

Supplementary Table S1.

## Data Availability

The validation dataset was available on request. Further information can be directed to the corresponding author. Data were obtained from the 2019 GBD of Disease database (https://vizhub.healthdata.org/gbd-results/). The GBD data set was hosted by the Institute for Health Metrics and Evaluation at Washington University. This public link to the database of GBD study is open, and the use of data does not require additional consent from IHME.
