# Supplementary material for: Time trends in the mortality of testicular cancer across the BRICS: an age-period-cohort analysis for the GBD 2019
Source: Sci Rep. 2024 Jun 3;14:12740. doi: 10.1038/s41598-024-63191-9 (PMC11148183; doi:10.1038/s41598-024-63191-9)
Supplement: Supplementary file 1 — Supplementary Table S1. [file 41598_2024_63191_MOESM1_ESM.docx]

**Table S1.** The lexis diagram of GBD data for the APC model

| Period (median) | Age groups | | | | | | | | | | | | Birth cohort (median) |
| --- | --- | --- | --- | --- | --- | --- | --- | --- | --- | --- | --- | --- | --- |
|  | 20-  24 | 25-  29 | 30-  34 | 35-  39 | 40-  44 | 45-  49 | 50-  54 | 55-  59 | 60-  64 | 65-  69 | 70-  74 | 75-  79 |  |
|  |  |  |  |  |  |  |  |  |  |  |  | X | 1911-1919 (1915) |
|  |  |  |  |  |  |  |  |  |  |  | X | X | 1916-1924 (1920) |
|  |  |  |  |  |  |  |  |  |  | X | X | X | 1921-1929 (1925) |
|  |  |  |  |  |  |  |  |  | X | X | X | X | 1926-1934 (1930) |
|  |  |  |  |  |  |  |  | X | X | X | X | X | 1931-1939 (1935) |
|  |  |  |  |  |  |  | X | X | X | X | X | X | 1936-1944 (1940) |
|  |  |  |  |  |  | X | X | X | X | X | X |  | 1941-1949 (1945) |
|  |  |  |  |  | X | X | X | X | X | X |  |  | 1946-1954 (1950) |
|  |  |  |  | X | X | X | X | X | X |  |  |  | 1951-1959 (1955) |
|  |  |  | X | X | X | X | X | X |  |  |  |  | 1956-1964 (1960) |
|  |  | X | X | X | X | X | X |  |  |  |  |  | 1961-1969 (1965) |
|  | **X** | X | X | X | X | X |  |  |  |  |  |  | 1966-1974 (1970) |
| 1990-1994 (1992) | X | X | X | X | X |  |  |  |  |  |  |  | 1971-1979 (1975) |
| 1995-1999 (1997) | X | X | X | X |  |  |  |  |  |  |  |  | 1976-1984 (1980) |
| 2000-2004 (2002) | X | X | X |  |  |  |  |  |  |  |  |  | 1981-1989 (1985) |
| 2005-2009 (2007) | X | X |  |  |  |  |  |  |  |  |  |  | 1986-1994 (1990) |
| 2010-2014 (2012) | X |  |  |  |  |  |  |  |  |  |  |  | 1991-1999 (1995) |
| 2015-2019 (2017) |  |  |  |  |  |  |  |  |  |  |  |  |  |

Note: X denotes mortality rate data of each age group from the corresponding period. For instance, the mortality rate of age 20-24 years in 1992 is filled in the square with a bold X (see table), and this square belongs to the cohort 1966-1974 (1970).
